# Supplementary figures and images for: Robotic High-Throughput Biomanufacturing and Functional Differentiation of Human Pluripotent Stem Cells
Source: bioRxiv. 2020 Aug 3:2020.08.03.235242. Preprint. [Version 1] doi: 10.1101/2020.08.03.235242 (PMC7418713; doi:10.1101/2020.08.03.235242)

Figure S1 (Tristan et al.)

A

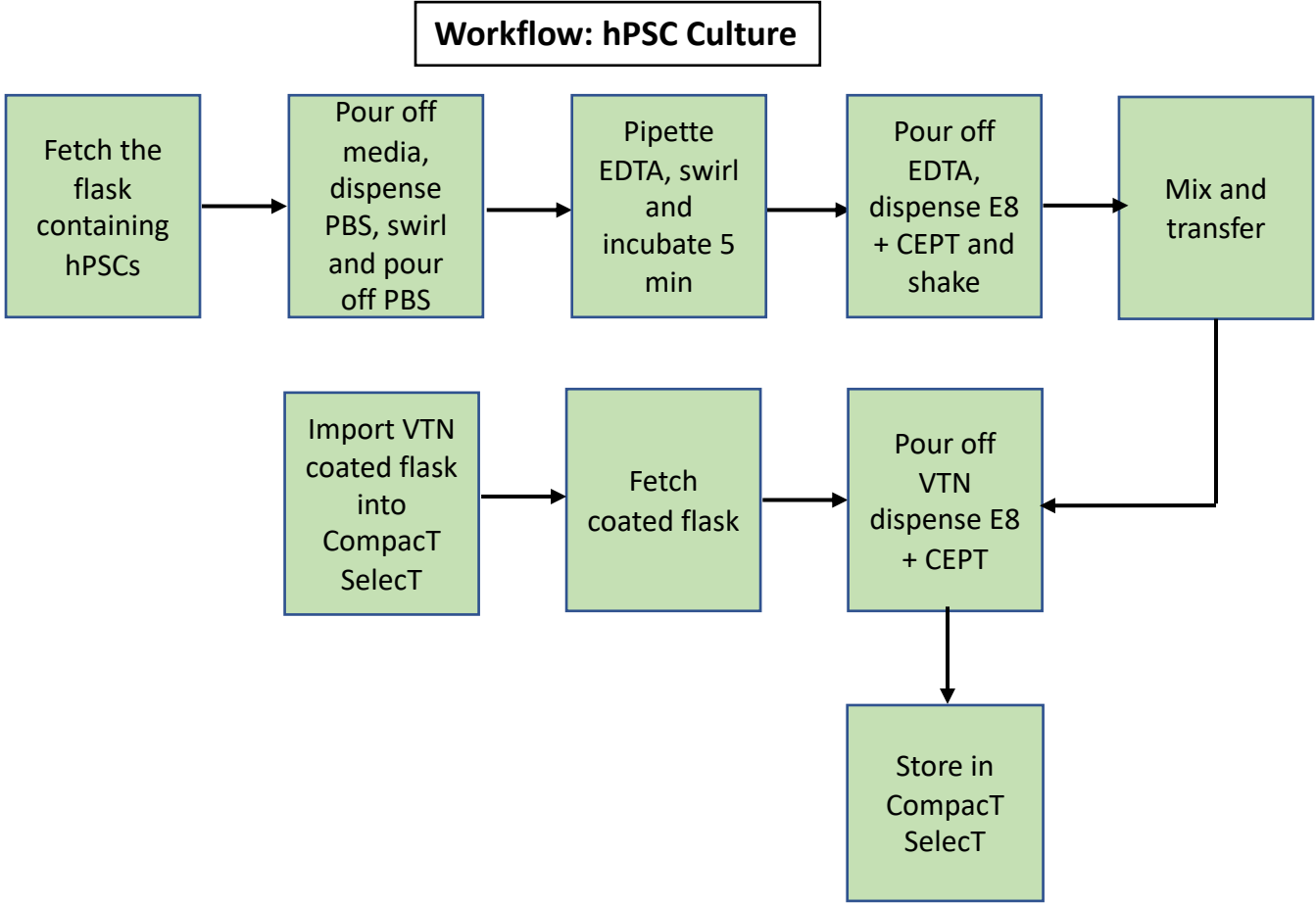

B

hESC (WA09)

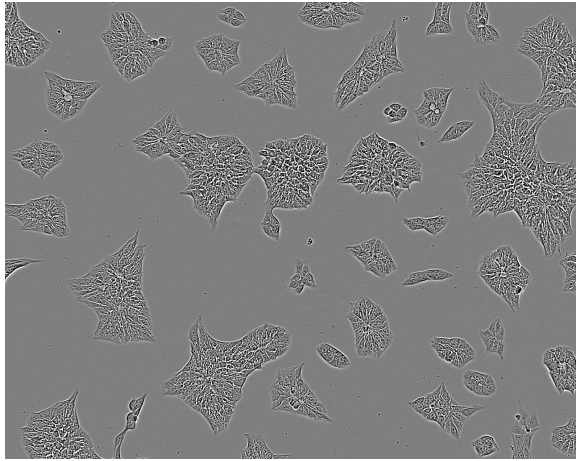

hiPSC (LiPSC-GR1.1)

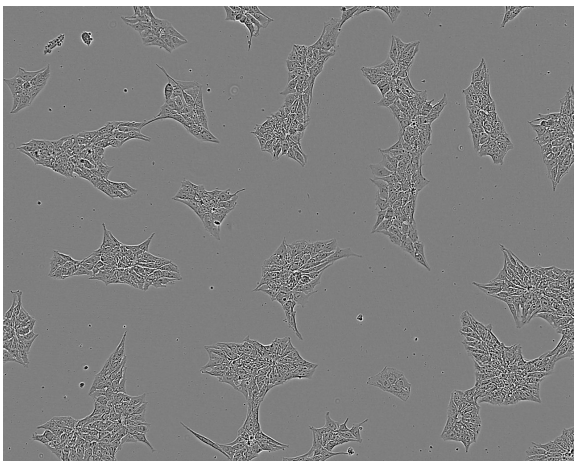

Supplement: Supplement 1 — Figure S1: Robotic Workflow for hPSC Culture (A) tandardized protocol developed for routine culture of hPSCs using CTST under chemically defined conditions. (B) Representative examples for robotically cultured hPSCs after passaging with the CEPT cocktail. Note the quality of cultures and absence of cellular debris at 24 h post-passage. [file media-1.pdf]

Figure S2 (Tristan et al.)

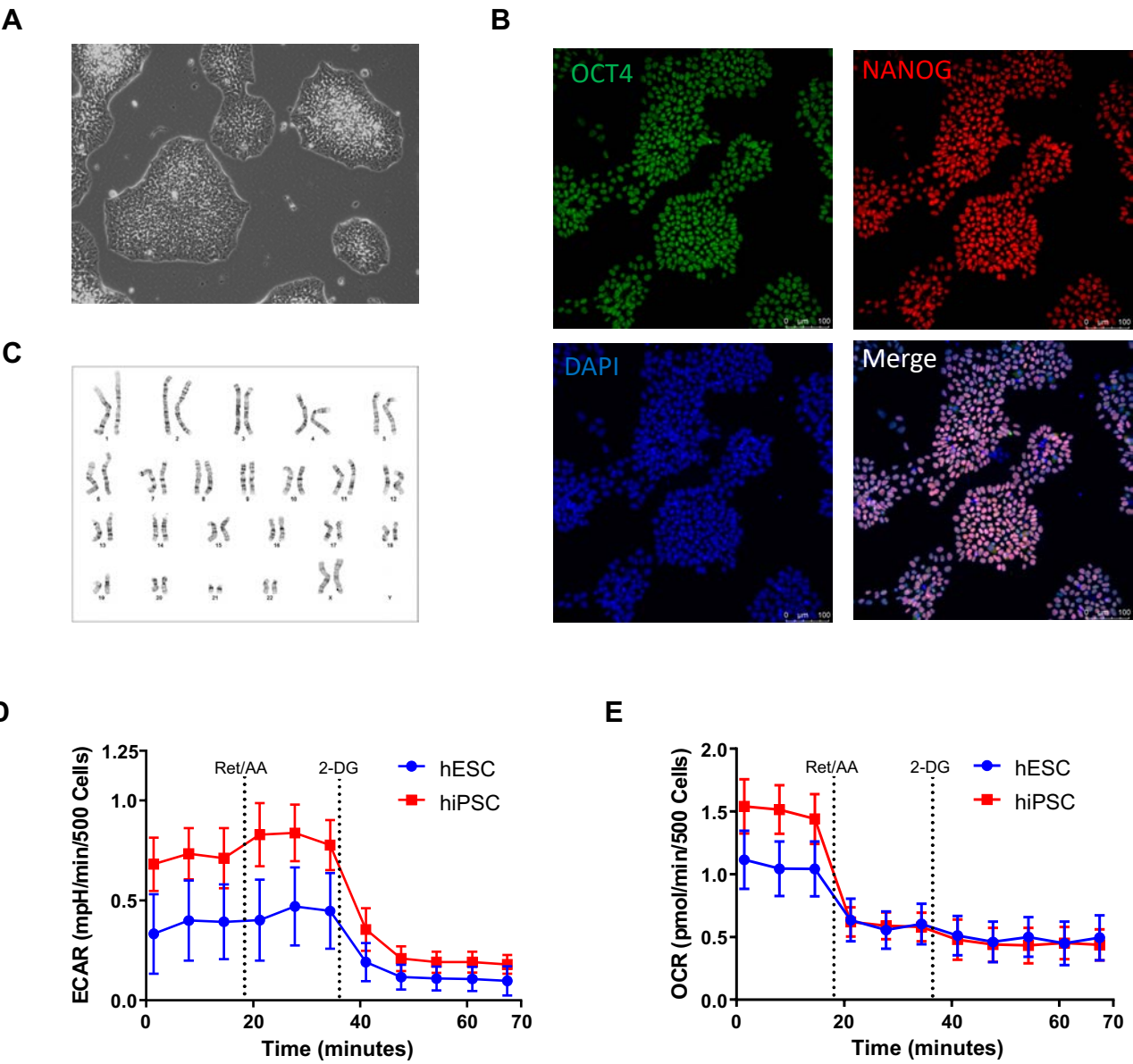

Supplement: Supplement 2 — Figure S2: Characterization of hESCs (WA09) Cultured by CTST (A) Representative overview of pluripotent stem cell colonies (Original magnification, 5x). (B) Immunocytochemical analysis showing expression of pluripotency-associated markers OCT4 and NANOG (magnification, 5x). (C) Cultured cells maintain a normal karyotype (passage 43). (D) Agilent Seahorse XF Glycolysis Rate Assay profile shows the extracellular acidification rate (ECAR) of hESCs and hiPSCs maintained by CTST. Serial injections of metabolic modulators (Ret/AA and 2-deoxyglucose [2-DG]) were performed at indicated time points. (E) Agilent Seahorse XF Glycolysis Rate Assay profile shows the oxygen consumption rate (OCR) of hESCs and hiPSCs maintained by CTST. Serial injections of metabolic modulators (Ret/AA and 2-deoxyglucose [2-DG]) were performed at indicated time points. [file media-2.pdf]

Figure S3 (Tristan et al.)

A

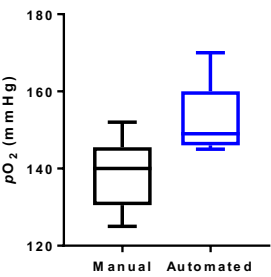

B

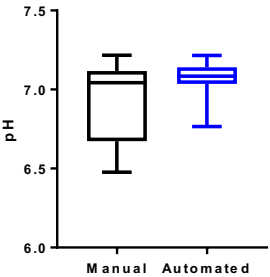

C

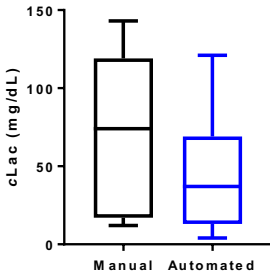

D

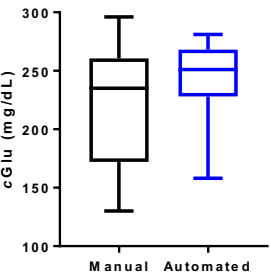

E

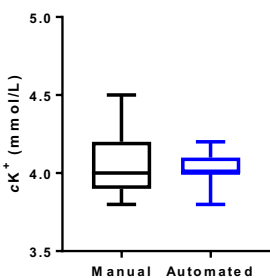

F

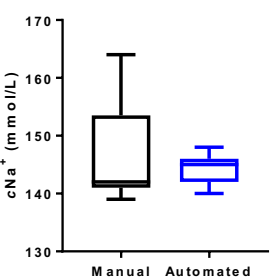

G

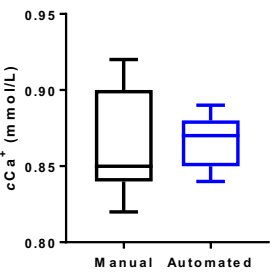

Supplement: Supplement 3 — Figure S3: Comparison of Manual and Automated Culture of hESCs (WA09) (A-G) Supernatants of cultures maintained either manually or by automation were analyzed by using the Vi-Cell MetaFLEX Bioanalyte Analyzer (Beckman). Box plots show the variation of fresh and spent media. See also Figures 2I–O. [file media-3.pdf]

Figure S6 (Tristan et al.)

A

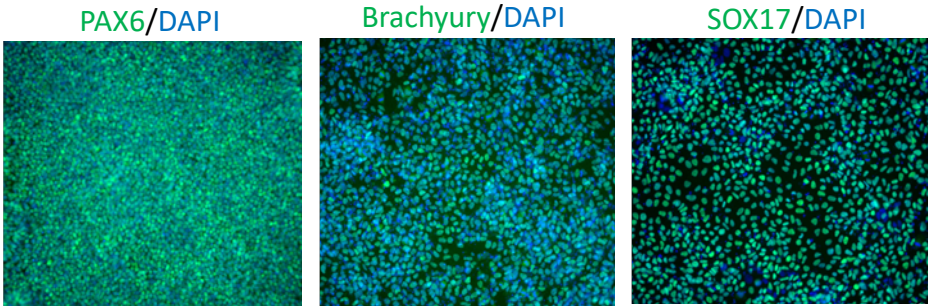

B

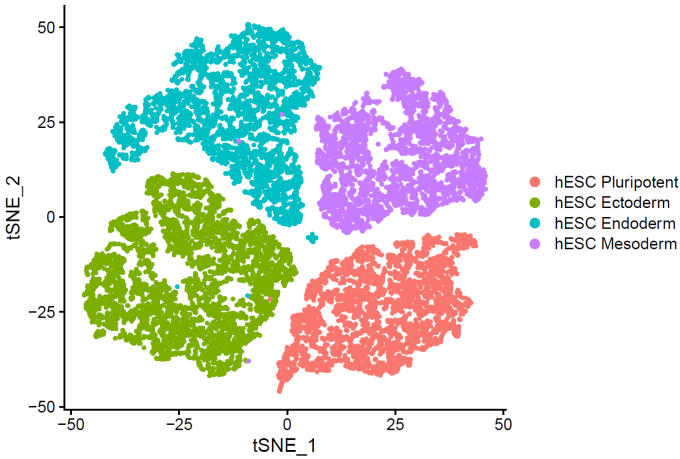

C

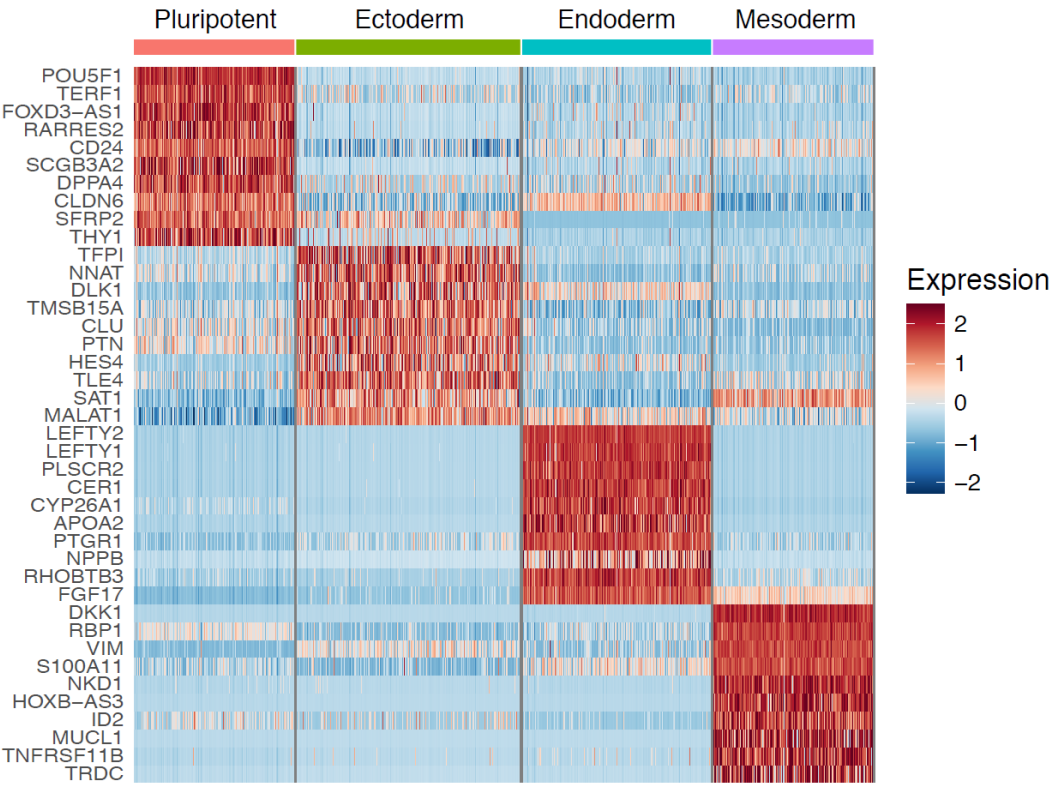

Supplement: Supplement 6 — Figure S6: Controlled Multi-Lineage Differentiation of hESCs (WA09) by CTST (A) Immunocytochemical analysis showing that large numbers of ectodermal (PAX6), endodermal (SOX17), and mesodermal (Brachyury) cells can be generated by CTST (magnification, 20x). (B) Single-cell analysis (RNA-seq) of pluripotent and differentiated cultures. (C) Heatmap showing efficient differentiation and cell type-specific expression of distinct genes in pluripotent and differentiated cells. [file media-6.pdf]

Figure S7 (Tristan et al.)

Day 10

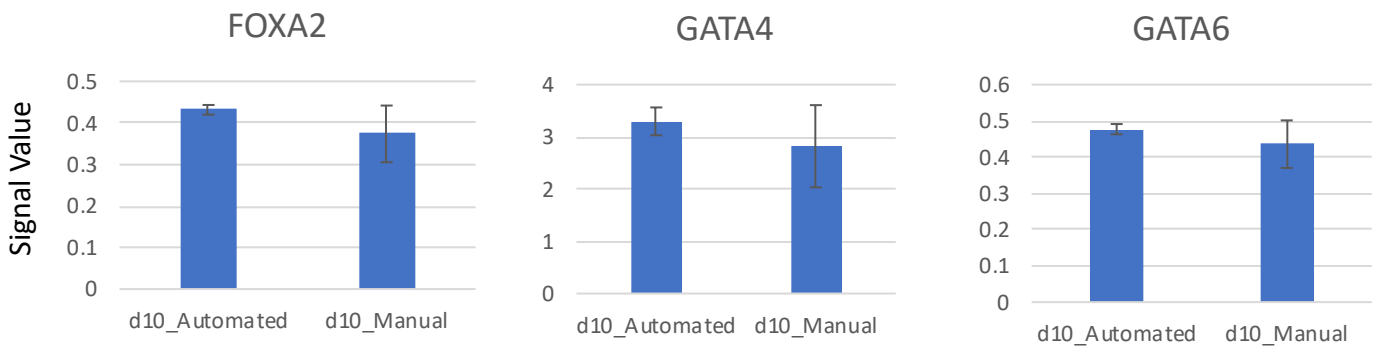

Day 20

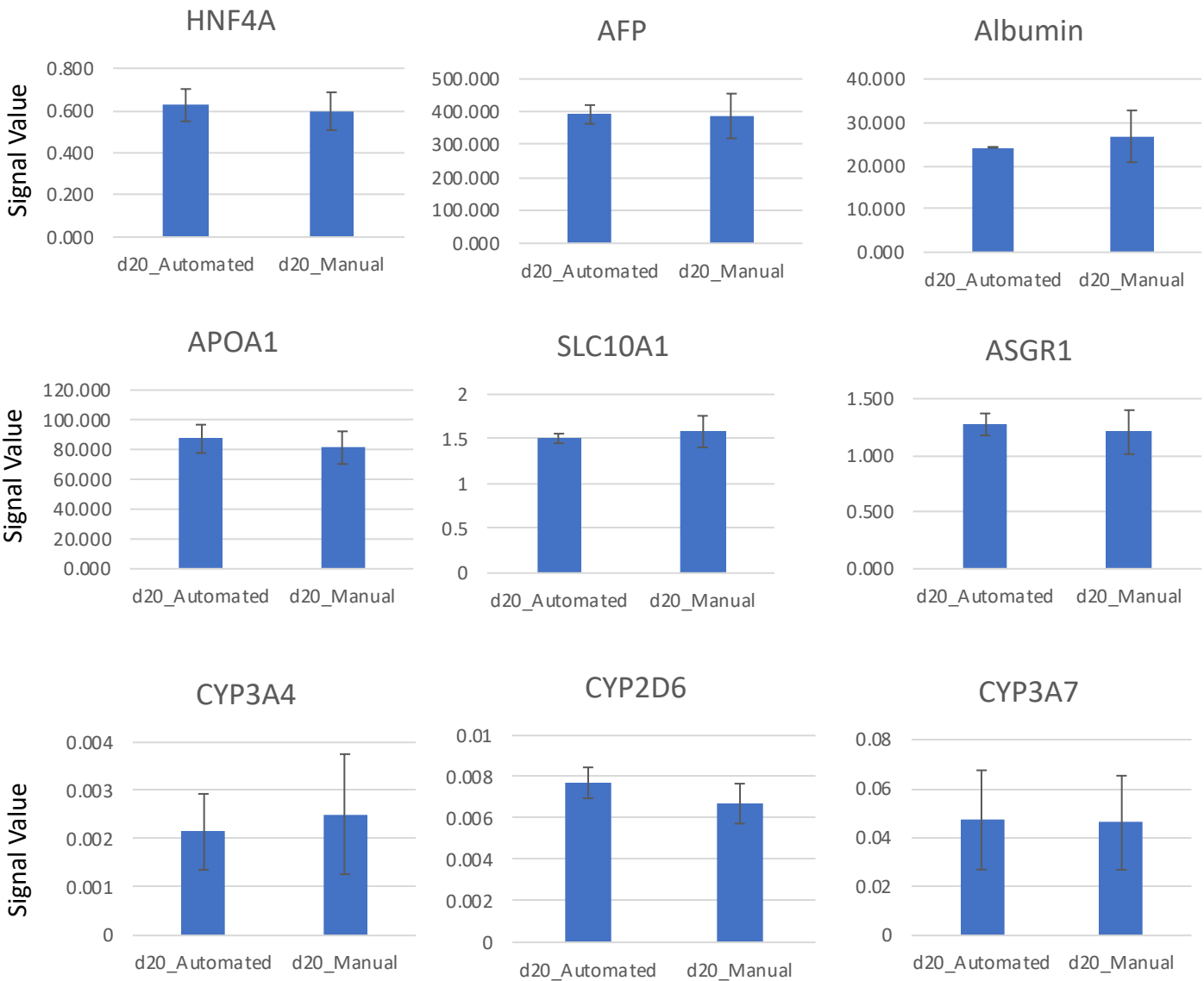

Supplement: Supplement 7 — Figure S7: RT-PCR Analysis and Comparison of Hepatocytes Differentiated Manually or Robotically Expression of typical endodermal and hepatocyte-specific genes at day 10 and 20. Note that virtually all genes tested are expressed at similar levels irrespective of manual or automated differentiation. [file media-7.pdf]

Figure S8 (Tristan et al.)

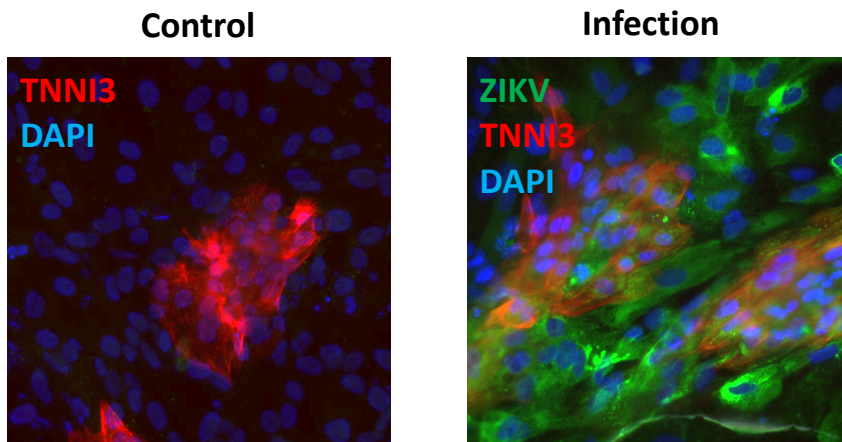

Supplement: Supplement 8 — Figure S8: Robotically Generated Cardiomyocytes Are Susceptible to ZIKV Infection Cardiomyocytes were derived from hiPSCs and exposed to ZIKV for 24 h. A specific antibody against flavivirus antigen shows that cells expressing cardiac troponin (TMMI3) can be infected by ZIKV (magnification, 40x). [file media-8.pdf]

**Figure S9 (Tristan et al.)**

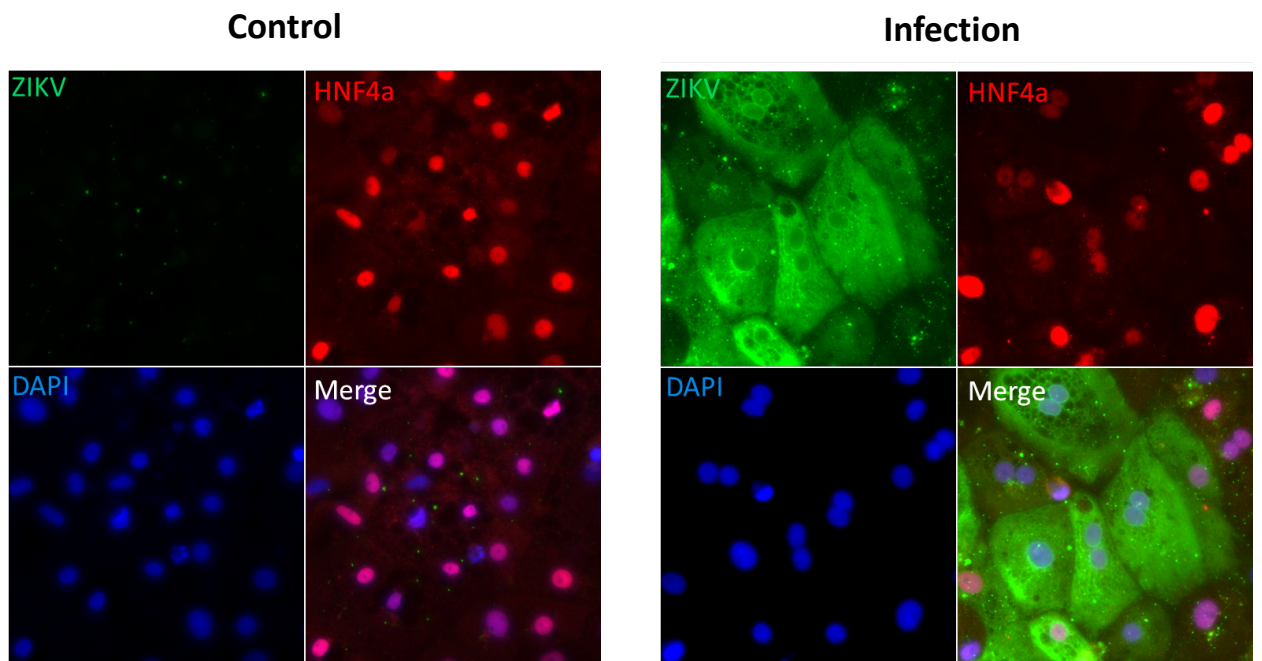

Supplement: Supplement 9 — Figure S9: Robotically Generated Hepatocytes Are Susceptible to ZIKV Infection Hepatocytes were derived from hiPSCs and exposed to ZIKV for 24 h. A specific antibody against flavivirus antigen shows that cells expressing HNF4A can be infected by ZIKV (magnification, 40x). [file media-9.pdf]
