# Supplementary material for: Robotic High-Throughput Biomanufacturing and Functional Differentiation of Human Pluripotent Stem Cells
Source: bioRxiv. 2020 Aug 3:2020.08.03.235242. Preprint. [Version 1] doi: 10.1101/2020.08.03.235242 (PMC7418713; doi:10.1101/2020.08.03.235242)
Supplement: Supplement 4 — Figure S4: Comparison the Efficiency of Robotic and Manual Cell Culture Automated versus manual cell culture features can be compared considering different plate formats, speed of media changes, and number of possible media changes based on the scenario that automation allows non-stop 24 h cell culture work, whereas manual cell culture is performed during an 8 h workday. In addition, while manual cell culture is typically done in 6-well plates, the CTST system can handle various flask and plate formats listed here. [file media-4.pdf]

Figure S4 (Tristan et al.)

Vessel	Surface Area Per Well (cm^2)	Total Vessel Surface Area (cm^2)	CompacT SelecT Capacity	Total Surface Area (cm^2)	Media Change Speed (min)	Media Changes Per Day	Manual Media Changes Per Shift (8h)
T175 Flask	175	175	90	15750	2	720	240
T75 Flask	75	75	90	6750	2	720	240
T175 Triple Flask	525	525	90	47250	2	720	240
6-well Plate	9.5	57	190	10830	6	240	80
24-well Plate	1.9	45.6	190	8664	6	240	80
96-well Plate	0.32	30.72	280	8602	6	240	80
384-well Plate	0.056	21.504	280	6021	6	240	80
