# Supplementary material for: Robotic High-Throughput Biomanufacturing and Functional Differentiation of Human Pluripotent Stem Cells
Source: bioRxiv. 2020 Aug 3:2020.08.03.235242. Preprint. [Version 1] doi: 10.1101/2020.08.03.235242 (PMC7418713; doi:10.1101/2020.08.03.235242)
Supplement: Supplement 5 — Figure S5: Robotic Workflow for Embryoid Body (EB) Formation (A) Protocol established for scalable production of EBs by using the CTST system under chemically defined conditions. (B) Representative phase-contrast image of robotically generated EBs, which can be cultured and scaled up in large T175 flasks (magnification, 5x). generated by the robotic cell culture. (C) ScoreCard analysis of EBs generated manually or robotically from hESCs and hiPSCs show similar differentiation potential into the three germ layers. [file media-5.pdf]

Figure S5 (Tristan et al.)

A

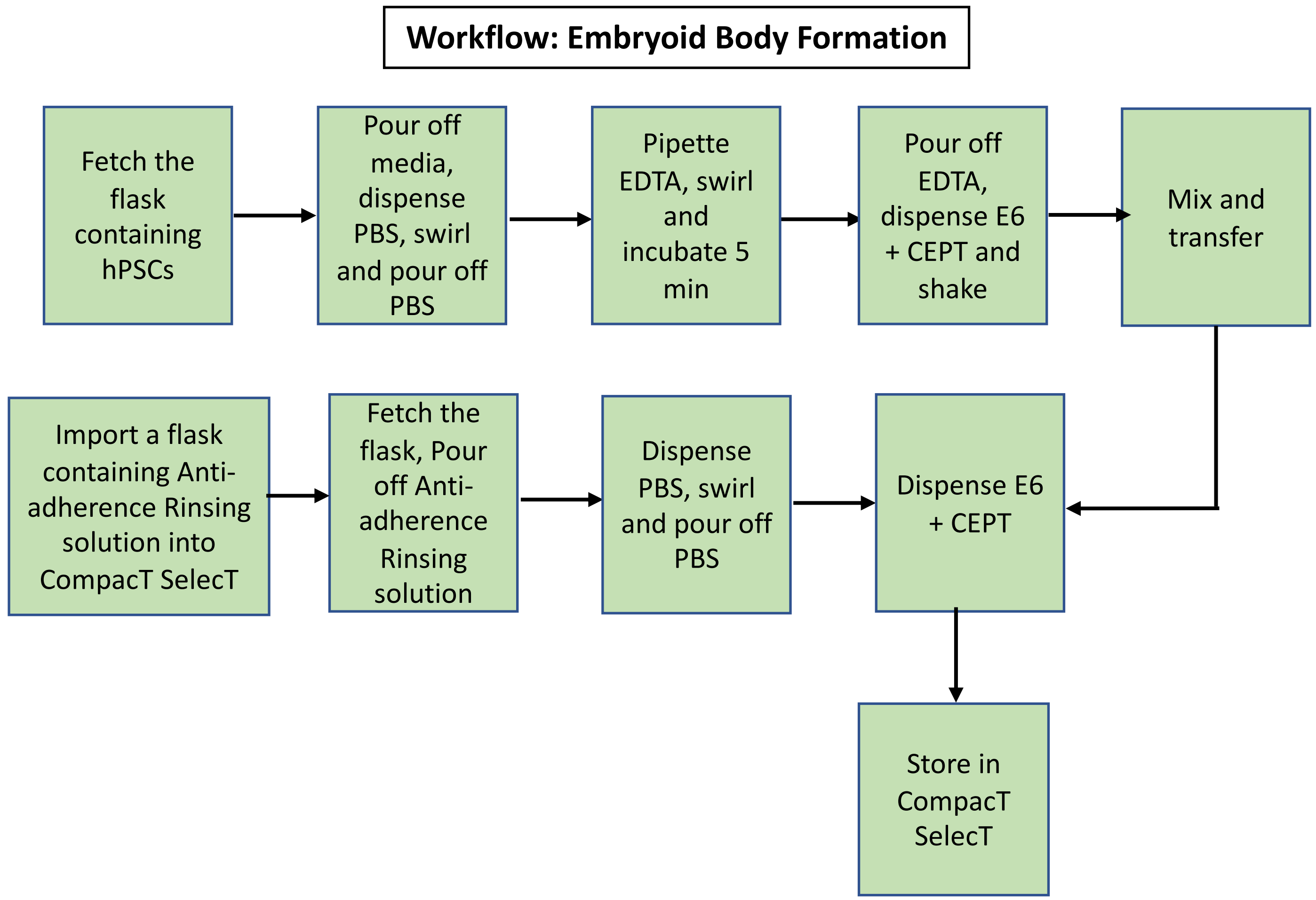

B

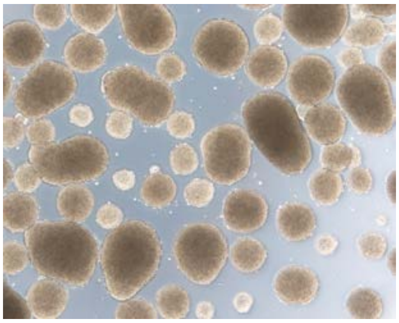

C

Sample Name	Self-renewal	Ectoderm	Mesoderm	Endoderm
hESC EB Manual	-5.05	1.30	3.07	0.56
hESC EB Automated	-4.74	1.24	2.58	0.16
hiPSC EB Manual	-0.80	0.29	0.13	-0.69
hiPSC EB Automated	0.18	0.63	0.23	-0.51

Gene expression relative to the reference standard

Upregulated						Downregulated
x > 1.5	1.0 < x ≤ 1.5	0.5 < x ≤ 1.0	-0.5 ≤ x ≤ 0.5	-1.0 ≤ x < -0.5	-1.5 ≤ x < -1.0	x < -1.5
