# Supplementary material for: Robotic High-Throughput Biomanufacturing and Functional Differentiation of Human Pluripotent Stem Cells
Source: bioRxiv. 2020 Aug 3:2020.08.03.235242. Preprint. [Version 1] doi: 10.1101/2020.08.03.235242 (PMC7418713; doi:10.1101/2020.08.03.235242)
Supplement: Supplement 12 — Table S3. User-Friendly and Scalable Production of Different Cell Types by CTST Depending on experimental needs, various cell types can be derived from hPSCs and scale-up production in different cell culture vessels. [file media-12.pdf]

Table S3 (Tristan et al.)

	Initial (Million)	Final (Million)	Scale-up per Plate or Flask (Million)						
Cell Type	Cells/cm²	Cells/cm²	384-well	96-well	24-well	6-well	T75	T175	T175 Triple
Ectoderm	0.10	0.9	19.4	26.65	41.04	51.30	67.50	157.50	472.50
Mesoderm	0.05	0.45	9.66	13.82	20.52	25.65	33.75	78.75	236.25
Endoderm	0.20	0.40	8.60	12.29	18.24	22.80	30.00	70.00	210.00
Hepatocytes	0.10	0.30	6.45	9.22	13.68	17.10	22.50	52.50	157.50
Cardiomyocytes	0.09	0.10	2.15	3.07	4.56	5.70	7.50	17.50	52.50
Neurons	0.05	0.43	9.30	13.21	19.61	24.51	32.25	75.25	225.75
