# Supplementary material for: Robotic High-Throughput Biomanufacturing and Functional Differentiation of Human Pluripotent Stem Cells
Source: bioRxiv. 2020 Aug 3:2020.08.03.235242. Preprint. [Version 1] doi: 10.1101/2020.08.03.235242 (PMC7418713; doi:10.1101/2020.08.03.235242)
Supplement: Supplement 13 — Table S4. Overview and Comparison of Published Papers and the Present Study Utilizing the CTST. Note the various advantages of the present study as compared to previous reports including the use of chemically defined media, enzyme-free passaging, and more extensive analysis and characterization of cells generated by automation. [file media-13.pdf]

Table S4 (Tristan et al.)

Reference	Automated System	Culture Medium for hPSCs	Coating Substrate	Passaging Reagent	Differentiation	Automated Scalability	Chemically Defined	Analysis
**Thomas et al., 2009**	CompacT SelecT	MEF-Conditioned Medium	Matrigel	Trypsin	Manual<br>Embryoid bodies<br>Cardiomyocytes	Partial	No	Pluripotency markers<br>Karyotype<br>MEA
**McLaren et al., 2013**	CompacT SelecT	N/A	PLO-Laminin	Trypsin	Automated<br>Lt-NES	Partial	N/A	Neural markers
**Soares et al., 2014**	CompacT SelecT	CDM-PVA	Porcine gelatin-MEF/FBS	Collagenase IV, Dispase	Manual<br>Multilineage	Partial	No	Pluripotency markers<br>qPCR
**Tristan et al., present study**	CompacT SelecT	E8 Medium (chemically defined)	Recombinant Vitronectin (chemically defined)	EDTA (enzyme-free)	Automated<br>Monolayer<br>Multi-Lineage<br>Embryoid bodies<br>Neurospheres<br>Cortical Neurons<br>Cardiomyocytes<br>Hepatocytes<br>Others (not shown in present study)	Full	Yes	Comparison manual vs. robotic<br>Pluripotency markers<br>Karyotype<br>Scorecard/qPCR<br>Bulk culture RNA-seq<br>Single-cell RNA-Seq,<br>Mass cytometry<br>Metabolic analysis<br>Robotic MEA<br>Disease modeling<br>High-throughput screening
