# Supplementary material for: Robotic High-Throughput Biomanufacturing and Functional Differentiation of Human Pluripotent Stem Cells
Source: bioRxiv. 2020 Aug 3:2020.08.03.235242. Preprint. [Version 1] doi: 10.1101/2020.08.03.235242 (PMC7418713; doi:10.1101/2020.08.03.235242)
Supplement: Supplement 15 — Method Table S1. Helios Panel. A CyTOF antibody panel against 28 targets for pluripotency, DNA damage, apoptosis and stress-signaling pathways. [file media-15.pdf]

Methods Table S1 (Tristan et al.)

Helios Panel: 28 Targets

Target	Label	Process
pS6 [S235/S236]	175Lu	AKT/mTOR/pS6 signaling - stressors
Caspase 3 (Cleaved)	142Nd	Apoptosis
Caspase 7 (Cleaved)	152Sm	Apoptosis
pBad	161Dy	Apoptosis
CyclinA	158Gd	Cell cycle
S-Phase (IdU)	127I	Cell cycle
CD278/ICOS	151Eu	Cell surface marker
pHistone H2A.X [Ser139]	147Sm	DNA damage
p53	143Nd	DNA repair/cell cycle/apoptosis
Stat3	173Yb	JAK/STAT signaling
pERK 1/2 [T202/Y204]	171Yb	MAPK ERK signaling
pMAPKAPK2 [T334]	159Tb	MAPK signaling
p-p38 [T180/Y182]	156Gd	MAPK signaling - stressors
IkBa	164Dy	NFkB signaling
Thioredoxin	146Nd	Oxidative Stress
CD44	162Dy	Pluripotency
Oct-3/4	165Ho	Pluripotency
Sox2	150Nd	Pluripotency
CD15 (SSEA-1)	144Nd	***Pluripotency***
Nanog	169Tm	Pluripotency
LCK	153Eu	***Pluripotency***
c-Myc	176Yb	Pluripotency
TRA-1-60	148Nd	Pluripotency
CD326 (EpCAM)	141Pr	***Pluripotency***
Ki-67	168Er	Proliferation
CD9	172Yb	RNA-Seq (pluripotency?)
CD24	166Er	RNA-Seq (pluripotency?)
CD81	145Nd	RNA-Seq (pluripotency?)
