# Supplementary material for: Robotic High-Throughput Biomanufacturing and Functional Differentiation of Human Pluripotent Stem Cells
Source: bioRxiv. 2020 Aug 3:2020.08.03.235242. Preprint. [Version 1] doi: 10.1101/2020.08.03.235242 (PMC7418713; doi:10.1101/2020.08.03.235242)
Supplement: Supplement 16 — Methods Table S2. TaqMan probes. List of TaqMan probes used for RT-qPCR. [file media-16.pdf]

Methods Table S2 (Tristan et al.)

RT-qPCR TaqMan Assays: 13 Gene Targets

Gene Query	Assay ID	Primer 1	Primer 2	Probe
FOXA2	Hs.PT.58.26032236	TGTTCATGCCGTTCATCCC	GGAGCGGTGAAGATGGAAG	TCCGACTGGAGCAGCTACTATGCA
GATA4	Hs.PT.58.259457	TTGCTGGAGTTGCTGGAA	GGAAGCCCAAGAACCTGAA	CCTGAAGGAGCTGCTGGTGTCTT
GATA6	Hs.PT.58.38396504	CCATCTTGACCCGAATACTTGA	GCAAAAATACTTCCCCCACAAC	TGCTCTCTCCCGCACCAGTC
HNF4A	Hs.PT.58.22303533	GATGTAGTCCTCCAAGCTCAC	GCCATCATCTTCTTTGACCCA	AAGATCAAGCGGCTGCGTTCC
Albumin	Hs.PT.56a.1501965	CAACAGAGGTTTTTCACAGCAT	GAGATCTGCTTGAATGTGCTG	AGATATACTTGGCAAGGTCCGCCC
AFP	Hs.PT.56a.571602	TCTGCATGAATTATACATTGACCAC	AGGAGATGTGCTGGATTGTC	AATGCTGCAAACTGACCACGCTG
SLC10A1	Hs.PT.58.40490059.g	ACTGGCTTTCAGAATTGCTTTG	GCTGCCACAACTGAAGAAAC	CCCTTTGTAGGTGCCATTTCCCAGA
APOA1	Hs.PT.56a.2455018.g	CTTTGAGCACATCCACGTACA	GCCGTGCTCTTCCTGAC	CTGCCAGAAATGCCGAGCCTG
ASGR1	Hs.PT.56a.24725395	CAGGCTGGAGTGATCTTCA	TTCAGCAACTTCACAGCGA	TCTTTCTTCCCACATTGCCTCCCTG
CYP3A4	Hs.PT.58.1272782	ATCATGTCAGGATCTGTGATAGC	GGGAAATATTTTGTCCTACCATAAGG	TGTTGACCATCATAAAAGCCCCACACT
CYP2D6	Hs.PT.58.45336286.g	CATACCTGCCTCACTACCAAA	TGTCCTGCCTGGTCCTC	CCAGGTGTGTCCAGAGGAGCC
CYP3A7	Hs.PT.58.26873929.g	CTATACAGACCATGAGAGAGCAC	CAGAACACCAGAGACCTCAA	CAGCACATTGGATGAAGCCCGTC
RPL13A	Hs.PT.58.45725862	CTCGACCATCAAGCACCAG	GCCGCCCCTGTTTCAAG	AGAAACCCTGCGACAAAACCTCCT
